# Intragingival injection of *Porphyromonas gingivalis*-derived lipopolysaccharide induces a transient increase in gingival tumour necrosis factor-α, but not interleukin-6, in anaesthetised rats

**DOI:** 10.1038/ijos.2015.9

**Published:** 2015-06-05

**Authors:** Hiroko Taguchi, Yuri Aono, Takayuki Kawato, Masatake Asano, Noriyoshi Shimizu, Tadashi Saigusa

**Affiliations:** 1 Department of Orthodontics, Nihon University School of Dentistry, Tokyo, Japan; 2 Department of Pharmacology, Nihon University School of Dentistry at Matsudo, Chiba, Japan; 3 Department of Pharmacology, Nihon University School of Dentistry, Tokyo, Japan; 4 Department of Oral Health Sciences, Nihon University School of Dentistry, Tokyo, Japan; 5 Department of Pathology, Nihon University School of Dentistry, Tokyo, Japan

**Keywords:** *Porphyromonas gingivalis*, lipopolysaccharide, gingiva, tumour necrosis factor-α, microdialysis

## Abstract

This study used *in vivo* microdialysis to examine the effects of intragingival application of lipopolysaccharide (LPS) derived from *Porphyromonas gingivalis* (*Pg*-LPS) on gingival tumour necrosis factor (TNF)-α and interleukin (IL)-6 levels in rats. A microdialysis probe with an injection needle attached to the surface of the dialysis membrane was implanted into the gingiva of the upper incisor. For comparison, the effects of LPS derived from *Escherichia coli* (*Ec*-LPS) on IL-6 and TNF-α levels were also analysed. *Pg*-LPS (1 μg/1 μL) or *Ec*-LPS (1 or 6 μg/1 μL) was applied by microsyringe, with gingival dialysates collected every hour. Enzyme-linked immunosorbent assay (ELISA) revealed that gingival dialysates contained approximately 389 pg·mL^−1^ of IL-6 basally; basal TNF-α levels were lower than the detection limit of the ELISA. *Pg*-LPS failed to alter IL-6 levels but markedly increased TNF-α levels, which remained elevated for 2 h after treatment. Neither IL-6 nor TNF-α were affected by *Ec*-LPS. Reverse transcriptase-polymerase chain reaction (RT-PCR) analysis revealed that the gingiva expresses Toll-like receptor (TLR) 2 and TLR4 mRNA. Immunohistochemical examination showed that TLR2 and TLR4 are expressed by gingival epithelial cells. The present study provides *in vivo* evidence that locally applied *Pg*-LPS, but not *Ec*-LPS, into the gingiva transiently increases gingival TNF-α without affecting IL-6. The present results suggest that TLR2 but not TLR4 expressed on gingival epithelial cells may mediate the *Pg*-LPS-induced increase in gingival TNF-α in rats.

## Introduction

Periodontal disease is a chronic inflammation of the gingiva resulting in the destruction of periodontal tissue^1^ and is frequently caused by infection with gram-negative pathogens.^2^ Lipopolysaccharide (LPS), a component of the gram-negative bacterial cell wall, plays an important role in periodontal tissue destruction.^3^ Previous studies have indicated that intragingival injection of LPS could induce periodontal inflammation as well as bone resorption in experimental animals. Multiple injections of LPS derived from *Escherichia coli* (*Ec*) into the palatal gingiva sustained the inflammatory infiltrate and induced the expression of matrix metalloproteinase-13, which induces connective tissue degradation.^4,5^ Gingival and periodontal inflammation with inflammatory infiltrate, apical migration of the junctional epithelium, interdental bone loss and activation of osteoclasts were induced in rats injected with LPS derived from *Salmonella typhimurium* (*S. typhimurium*) into the gingiva.^6^ These findings clearly suggest that locally applied LPS can increase inflammatory mediators at the injection site.

In periodontal diseases, inflammatory cytokines such as the interleukins (IL) and tumour necrosis factor (TNF) may promote the degeneration of inflamed periodontal tissues.^7,8,9^ IL-6 and TNF-α are inflammatory cytokines produced after microbial recognition and are involved in the pathogenesis of periodontal disease.^10^ IL-6 is thought to be involved in inflammatory cell migration and osteoclastogenesis in periodontal disease.^11,12^ Thus, IL-6 could play a facilitative role in the pathogenesis of periodontal disease. TNF-α is known to induce alveolar bone resorption and the loss of connective tissue attachment.^11,13^ In addition to these direct effects on the bone and connective tissue, TNF-α has been reported to increase the production of pro-inflammatory cytokines such as IL-1β and IL-6.^11,14,15,16^ TNF-α has also been shown to suppress the progression of periodontal disease by increasing the migration of phagocytic cells to infected and inflamed sites.^10^ Thus, TNF-α can either facilitate or inhibit the pathogenesis of periodontal disease.^10^

In previous animal experiments, periodontal tissue destruction was observed at least 5–7 days after the onset of repeated injections of *Ec*-derived LPS (ref. ^5^) or after a single injection of *S. typhimurium*-derived LPS (ref. ^6^) into the gingiva. However, these reports only used LPS derived from microorganisms that do not commonly exist in clinically diseased periodontal tissue. The effects of intragingival injection of LPS on the inflammatory cytokines in the injection site immediately after the LPS treatment are also unknown. *Porphyromonas gingivalis* (*Pg*), a Gram-negative, rod-shaped, anaerobic bacterium, is a major contributor to the development of inflammatory periodontal disease,^2^ and its cell wall contains LPS.^17,18^ In the present study, we focally injected LPS derived from *Pg* (*Pg*-LPS) into the gingiva of rats anaesthetised with urethane and analysed its effects on the IL-6 and TNF-α levels in gingival dialysate collected from the injection sites by *in vivo* microdialysis. The *in vivo* microdialysis method can sampled continuously in a single animal. Furthermore, unlike whole tissue samples collected by dissection, *in vivo* microdialysis allows the extracellular fluid from a discrete area of the body to be collected through the dialysis membrane.^19,20^ Thus, in contrast to homogenised tissue samples, the gingival samples in the present study were (i) collected in a relatively limited area of the gingiva, and (ii) contain substances that were secreted into the extracellular space and diffused across the semi-permeable dialysis membrane according to the concentration gradient. A microdialysis probe equipped with an injection needle was used to examine the effects of intragingival injection of LPS on IL-6 and TNF-α levels at the injection site. For comparison, the effects of LPS derived from *Ec* (*Ec*-LPS) on the gingival levels of IL-6 and TNF-α were also examined, because previous *in vitro* studies have revealed differences in the effects of *Pg*-LPS and *Ec*-LPS on the expression of inflammatory cytokines, including IL-6 and TNF-α, in gingival epithelial cells,^21^ fibroblasts,^17,18^ periodontal ligament cells^22^ and macrophages.^17^ Next, the mRNA expression of Toll-like receptor (TLR)2 and TLR4, which act as LPS receptors,^23,24^ was confirmed in gingival tissue. Finally, the distribution of TLR2 and TLR4 in the periodontal tissue was characterised using immunohistochemistry.

## Material and Methods

### Animals

About 8- to 9-week-old male Sprague-Dawley rats (Takasugi Experimental Animals Supply, Saitama, Japan) weighing 300–350 g were used. The rats were kept at constant room temperature (RT; (23±2) °C) and relative humidity (55%±5%) under a 12-h day and night cycle (lights on 07:00), with free access to food and water.

The experiments were approved by the Animal Experimentation Committee of Nihon University School of Dentistry and were performed in accordance with national and international guidelines for the care and welfare of laboratory animals. All efforts were made to minimise animal suffering and to reduce the number of animals used.

### *In vivo* microdialysis

**Figure 1:**
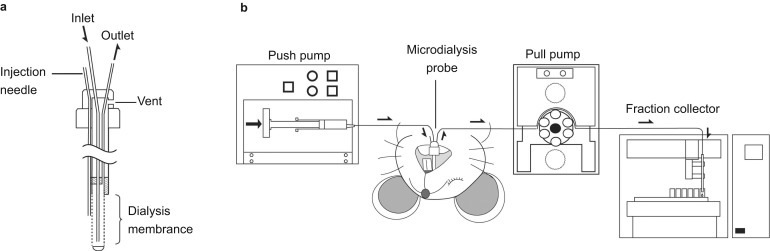
**Microdialysis system equipments.** (**a**) Microdialysis probe with a needle for drug microinjection. (**b**) Schematic illustration of the *in vivo* microdialysis system using a push-pull pump.

A commercially available I-shaped dialysis probe with a needle for drug microinjection was used (Figure 1a; PEMI-4.5-02; Eicom, Kyoto, Japan). Briefly, the dialysis probe consisted of a 4.5 mm shaft with a 2 mm length of polyethylene (PE) membrane with an outside diameter of 440 μm and a molecular weight cut-off of 1 000 kDa. The needle for drug microinjection was made with fused silica and had an outside diameter of 150 μm and an inside diameter of 75 μm. The needle was attached to the probe with its tip located just above (<40 μm) the surface of the centre of the dialysis membrane. The PE membrane of the dialysis probe was briefly conditioned with ethanol (<3 s) before implantation.

The animals were anaesthetised with urethane (1.5 g·kg^−1^ intraperitoneally (i.p.); Sigma-Aldrich, St. Louis, MO, USA) and their internal temperature was maintained at 36 °C with a thermostatistically controlled heating pad. The depth of anaesthesia was assessed by monitoring for the presence or absence of corneal reflexes and withdrawal responses from a noxious pinch of the hind paw. The edge of the gingiva of the right upper incisor was locally anaesthetised with 2% lidocaine, then the PE membrane of the microdialysis probe was manually implanted into the gingiva of the distal-palatal (upper right) incisor. The shaft of the probe was affixed to the edge of incisor using dental acrylic cement. The tip of the probe was placed approximately 3 mm below the edge of the gingiva; thus, the microdialysis membrane was inserted 2 mm into the gingiva.

The inlet and outlet of the dialysis probe were connected to fluorinated ethylene propylene (FEP) tubing (inside diameter, 250 μm), then perfusion was performed with a modified ringer solution (NaCl, 147 mmol·L^−1^; KCl, 4 mmol·L^−1^; CaCl_2_, 2.3 mmol·L^−1^; pH 7.4) that contained 0.15% bovine serum albumin (BSA) using a microsyringe push pump (EP-60; Eicom, Kyoto, Japan) together with a peristaltic pull pump (ERP-10; Eicom, Kyoto, Japan). These push and pull pumps operate simultaneously and allow the modified ringer solution to perfuse into the dialysis membrane at various flow rates (1–10 μL·min^−1^). The outflow was connected by FEP tubing to a temperature-controlled fraction collector system (EFC-82; Eicom, Kyoto, Japan; Figure 1b). To obtain stable baseline recordings, the probes were perfused with the modified ringer solution for 3 h at a flow rate of 10 μL·min^−1^. Then, perfusate samples were taken every 60 min for 6 h at a flow rate of 1 μL·min^−1^. The samples were collected into polypropylene tubes and stored at 4 °C.

### Intragingival injection of LPS

The forms of LPS used in this study were *Pg*-LPS (*P. gingivalis* LPS; InvivoGen, San Diego, CA, USA) and *Ec*-LPS (055:B5; Sigma-Aldrich, St. Louis, MO, USA). The strain of *Ec*-LPS was selected based on previous reports.^5,25^

For intragingival injection, *Pg*-LPS or *Ec*-LPS in sterile endotoxin-free water was manually injected in a volume of 1.0 μL over a 30-s period with a microsyringe (Hamilton, Reno, NV, USA). The syringe was left *in situ* for 30 s after each injection.^26^ The control group received an injection of the vehicle. The injection volume (1 μL) was chosen to minimise the diffusion of the LPS based on the report from Dumitrescu *et al.*^6^ Doses of *Pg*-LPS (1 μg) and *Ec*-LPS (1–6 μg) were based on the manufacturers' instructions. To avoid non-specific effects, doses of *Pg*-LPS higher than 1 μg/1 μL and doses of *Ec*-LPS higher than 6 μg/1 μL were not used for intragingival injection.

### Enzyme-linked immunosorbent assay

The concentrations of IL-6 and TNF-α in gingival samples were determined using commercially available enzyme-linked immunosorbent assay (ELISA) kits (rat IL-6 and rat TNF-α; R&D Systems, Minneapolis, MD, USA), according to the manufacturer's instructions. The dialysate samples were stored at 4 °C in polypropylene tubes until ELISA testing and were analysed within 24 h of sampling. All ELISA samples for IL-6 and TNF-α were diluted in modified ringer solution. Sixty microlitres of microdialysed samples were collected and diluted with the same volume of modified ringer solution. Fifty microlitres of diluted samples were subjected to the ELISA. The absorbance was measured on a microplate reader (model 3550; Bio-Rad, Tokyo, Japan).

### Reverse transcriptase-polymerase chain reaction

The animals were anaesthetised with urethane (1.5 g·kg^−1^, i.p., Sigma-Aldrich, St. Louis, MO, USA) and the gingiva of the right upper incisor was anaesthetised locally with 2% lidocaine. Gingival tissue was recovered from the edge of gingiva of the right upper incisor under RNase-free conditions and transferred to tubes containing RNA stabilisation reagent (RNA later; Ambion, Austin, TX, USA). Total RNA was purified using the RNeasy mini kit (QIAGEN, Tokyo, Japan), and reverse transcription was done using random hexamers to generate cDNA. polymerase chain reaction (PCR) amplification was performed in a reaction mixture consisting of *EX Taq* (TaKaRa, Tokyo, Japan) and the required reagents, along with cDNA and PCR primer pairs for TLR2, TRL4 or the housekeeping gene glyceraldehyde-3-phosphate dehydrogenase (GAPDH), as a control. The amplification reaction was performed using My Cycler (Bio-Rad, Tokyo, Japan) with an initial denaturation at 95 °C for 3 min, followed by 40 cycles of 95 °C for 30 s, 60 °C for 30 s and 72 °C for 30 s. The primers used in this study were as follows: TLR2, 5′-GGCCACAGGACTCAAGAGCA-3′ (forward) and 5′-AGAGGCCTATCACAGCCATCAAG-C-3′ (reverse); TLR4, 5′-CTCACAACTTCAGTGGCTGGATTTA-3′ (forward) and 5′-GTCTCCACAGCCACCAGATTCTC-3′ (reverse); and GAPDH, 5′-CAAACAGGCCTGGGCACTA-3′ (forward) and 5′-TGGTAACCAGGCACCCAATAA-3′ (reverse). The resulting PCR products were subjected to 2% agarose gel electrophoresis and were visualised by ethidium bromide staining.

### Immunohistochemical staining

The animals were anaesthetised with urethane (1.5 g·kg^−1^, i.p., Sigma-Aldrich, St. Louis, MO, USA) and transcardially perfused with saline followed by a fixative containing 4% paraformaldehyde in 0.1 mol·L^−1^ phosphate buffer (pH 7.4).

Gingival samples were fixed in 10% formalin and embedded in paraffin. About 4 µm thick sections were prepared, deparaffinised in xylene and rehydrated with 100% ethanol. Endogenous peroxidase activity was inactivated with 0.3% hydrogen peroxide in methanol for 20 min at RT. The sections were boiled in 10 mmol·L^−1^ citrate buffer (pH 6.0) for 20 min and cooled. To block non-specific binding, the sections were incubated with 1% BSA in Tris-buffered saline (TBS) for 1 h at RT. The blocking solution was removed, and the slides were incubated with primary antibodies for 1 h at RT or overnight at 4 °C. The primary antibodies against TLR2 and TLR4 were purchased from Santa Cruz Biotechnology (Santa Cruz, CA, USA) and diluted 1 ∶ 100 with TBS containing 1% BSA (1% BSA–TBS). Negative control slides were incubated with 1% BSA–TBS instead of the primary antibody. The sections were then incubated with horseradish peroxidase (HRP)-conjugated anti-rabbit immunoglobulin G (IgG) (diluted 1 ∶ 500) (Jackson ImmunoResearch, West Grove, PA, USA) for 1 h at RT. After the sections were washed, they were developed with freshly prepared diaminobenzidine (DAB) chromogen solution (Sigma, Tokyo, Japan) for 7 min, counterstained with haematoxylin for 10 s, dehydrated in a series of ethanol dilutions, cleared in xylene and mounted on glass cover slips.

The images were viewed and photographed using a Leica DM5500B light microscopy system (Leica, Tokyo, Japan).

### Statistical analysis

Comparison of time-course data (0–240 min) was performed using two-way analysis of variance (ANOVA) for repeated measures with treatment and time (repeated) as factors, followed by a post-hoc Scheffé's test where appropriate. Statistical significance was considered to be *P*<0.05.

## Results

### Effects of intragingival injection of LPS on gingival IL-6 and TNF-α measured by *in vivo* microdialysis

**Figure 2:**
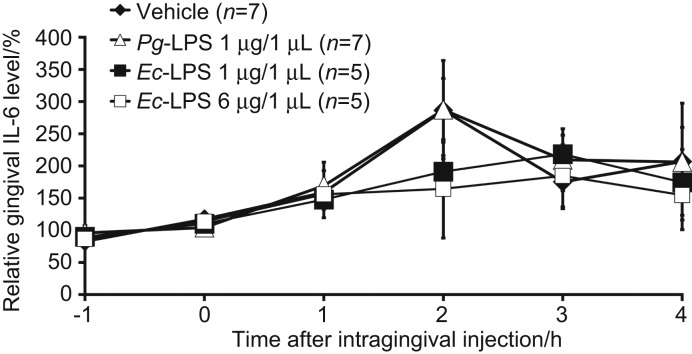
**Effects of intragingival injection of *Pg*-LPS or *Ec*-LPS on the gingival levels of IL-6.** All values are presented as a percentage of the baseline level of IL-6, which was the mean of the IL-6 level in the two samples measured immediately prior to LPS treatment. The data are expressed as the mean change in the 1-hr observation period (ordinate) after intragingival injection of LPS (abscissa). Vertical bars indicate SEM. The basal gingival IL-6 level is control (100%). Neither injection of *Pg*-LPS nor *Ec*-LPS into the gingiva-altered gingival levels of IL-6 (*n*=6–7). *Ec*-LPS, lipopolysaccharide derived from *Escherichia coli*; IL, interleukin; LPS, lipopolysaccharide; *Pg*-LPS, lipopolysaccharide derived from *Porphyromonas gingivalis*; SEM, standard error of the mean.

**Figure 3:**
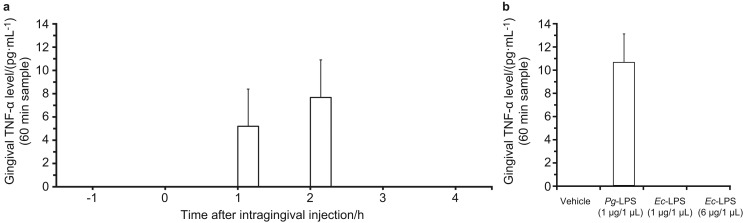
**Effects of intragingival injection of *Pg*-LPS or *Ec*-LPS on the gingival levels of TNF-α.** (**a**) Intragingival injection of *Pg*-LPS (1 *μ*g/1 *μ*L) induced a transient increase in gingival levels of TNF-α (*n*=5). Intragingival injection of *Ec*-LPS did not alter gingival levels of TNF-α. Since the basal levels of TNF-α were below the detection limit of the ELISA, the data are expressed as the mean absolute amount of TNF-α in gingival dialysates (pg·mL^−1^). (**b**) Average maximum increase in gingival levels of TNF-α induced by intragingival injection of *Pg*-LPS. The maximum increase in TNF-α in each rate was observed 1–3 h after the injection (*n*=5). *Ec*-LPS, lipopolysaccharide derived from *Escherichia coli*; *Pg*-LPS, lipopolysaccharide derived from *Porphyromonas gingivalis*; TNF, tumor necrosis factor.

Gingival dialysates contained an average of 389 pg·mL^−1^ IL-6 at baseline; basal TNF-α levels were lower than the detection limit of the ELISA kit (5 pg·mL^−1^) (Table 1). Neither *Pg*-LPS (1 μg) nor *Ec*-LPS (1 or 6 μg) affected IL-6 levels (Figure 2; two-way ANOVA, treatment: *F*_(3, 60)_=0.3, *P*=0.8). *Pg*-LPS (1 μg) induced a marked increase in TNF-α levels lasting 2 h after treatment (Figure 3; two-way ANOVA, treatment: *F*_(3, 66)_=11.0, *P*<0.05), but *Ec*-LPS (1 or 6 μg) failed to alter TNF-α levels (Figure 3a and 3b). A post-hoc Scheffé's test revealed that the effects of *Pg*-LPS (1 μg) differed significantly from the vehicle (*P*<0.05).

### Histological examination of the effects of intragingival injection of LPS on gingival tissue

Since a *Pg*-LPS (1 μg)-induced transient increase in gingival TNF-α was observed 1–2 h after the intragingival injection, histological analysis was performed on gingival sections obtained 1–2 h after the injection of LPS.

Light microscopy revealed that the intragingival injection of *Pg*-LPS (1 μg) or *Ec*-LPS (6 μg) failed to induce inflammatory histological changes in the periodontal tissue at the injection site (data not shown).

### TLR2 and TLR4 mRNA expression in the gingiva of the upper incisor

**Figure 4:**
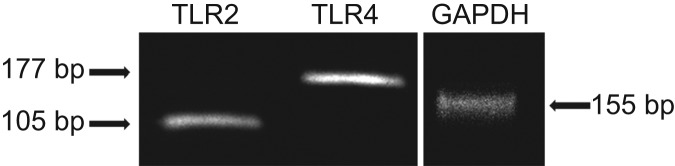
**RT-PCR analysis of expression of TLR2 and TLR4 mRNA in rat gingival tissue.** Total RNA was extracted from rat gingival tissue. After cDNA was generated, PCR was performed with specific primers against TLR2, TLR4 and GAPDH. The PCR products were loaded onto a 1.5% agarose gel and photographed. The expected PCR products of TLR-2, TLR-4 and GAPDH are 177, 105 and 155 bp, respectively. GAPDH, glyceraldehyde-3-phosphate dehydrogenase; PCR, polymerase chain reaction; RT-PCR, reverse transcriptase-polymerase chain reaction; TLR, Toll-like receptor.

RT-PCR analysis confirmed that TLR2 and TLR4 mRNA was expressed in the gingiva of the upper incisors of urethane-anaesthetised rats (Figure 4).

### Immunohistochemical staining for TLR2 and TLR4 in the periodontal tissue of the upper incisor

**Figure 5:**
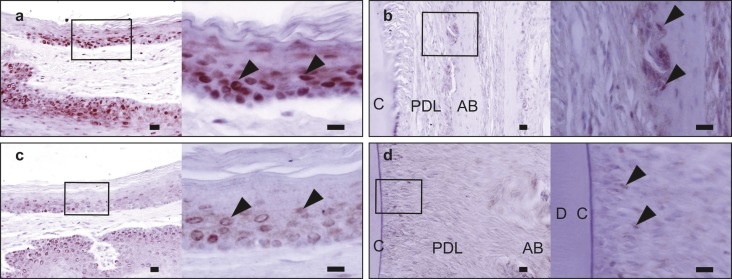
**Immunohistochemical staining for TLR2 and TLR4 in the periodontal tissue of the upper incisors.** A high magnification view of the boxed region in the left panel is shown in the right panel. TLR2 expression in epithelial cells (arrowheads in **a**); TLR2 expression in periodontal ligament fibroblasts (arrowheads in **b**); TLR4 expression in epithelial cells (arrowheads in **c**); and TLR4 expression in periodontal ligament fibroblasts (arrowheads in **d**). Bar = 20 μm. AB, alveolar bone; C, the cementum; D, dentine; PDL, periodontal ligament; TLR, Toll-like receptor.

TLR2 was expressed mainly in the basal and suprabasal cells of the oral stratified squamous epithelium (Figure 5a). In addition, TLR2 was also expressed in a few periodontal ligament fibroblasts (Figure 5b). When the staining intensity was compared between cell populations, TLR2 expression was much higher in epithelial cells than in periodontal ligament cells. TLR4 expression was also detected in the suprabasal cells of the epithelial layer, but it was not observed in the periodontal ligament cells (Figure 5c and 5d). Although TLR2 and TLR4 expression levels in the periodontal tissue of the upper incisor were not quantitatively determined, the overall staining intensity of TLR2 was higher than that of TLR4. Neither TLR2 nor TLR4 expression was detected in gingival fibroblasts. In agreement with previous observations,^27^ TLR2 and TLR4 were expressed in hair follicle cells, and as a control, expression of TLR4, but not TLR2, was confirmed in the muscle cells of the rat orofacial area (data not shown).

## Discussion

In the present study, we analysed the effects of focal application of *Pg*-LPS or *Ec*-LPS on IL-6 and TNF-α levels in the gingiva of the distal-palatal incisor. A microdialysis probe were inserted into the gingiva and extracellular fluid was collected through a semi-permeable membrane. The IL-6 and TNF-α concentrations in the gingival dialysates were determined by ELISA. Gingival dialysates contained a significant amount of basal IL-6 (approximately 389 pg·mL^−1^), but basal TNF-α levels were lower than the detection limit of the ELISA (5 pg·mL^−1^). Anaesthetics for laboratory animals have been shown to have a slight influence on the basal immune status. In particular, urethane increases the mRNA expression of IL-6 and decreases that of TNF-α in the rat spleen;^28^ therefore, the possible influence on levels of IL-6 and TNF-α in the gingival dialysate should be taken into account.

The present study revealed that intragingival injection of *Pg*-LPS did not alter IL-6 levels, but it transiently increased TNF-α in the gingiva. These results are in agreement with an earlier report that local injection of *Pg*-LPS into the rat paw induced a transient increase in TNF-α at the injection site.^29^ In contrast to *Pg*-LPS, intragingival application of *Ec*-LPS affected neither IL-6 nor TNF-α in the gingival dialysate. Various reports have suggested that *Pg*-LPS induces its effects by (i) stimulating TLR2,^23^ (ii) activating TLR2 and/or TLR4,^30^ or (iii) stimulating TLR4 but not TLR2.^31^ *Ec*-LPS is known to selectively activate TLR4, compared to *Pg*-LPS.^23,24^ It is suggested that *Ec*-LPS induces cytokines through TLR4-NF-κB and/or TLR4-p38MAPK and that *Pg*-LPS produces cytokines via TLR2-JNK.^32^ In the present study, gingival tissue obtained from the distal portion of the upper right incisor of anaesthetised rats contained both mRNA for TLR2 and TLR4. Moreover, immunohistochemical examination revealed that the gingival epithelial cells, but not gingival fibroblasts, expressed TLR2 and TLR4 at the protein level. Since intragingival application of *Pg*-LPS, but not *Ec*-LPS, increased gingival levels of TNF-α, these results suggest that TLR2, but not TLR4, in gingival epithelial cells might mediate the *Pg*-LPS-induced TNF-α secretion. In fact, *Pg*-LPS has been shown to induce the production of TNF-α from cultured gingival epithelial cells from experimental animals as well as humans (mouse;^33^ human^34,35^).

In the present study, we analysed the effects of *Pg*-LPS and *Ec*-LPS on the levels of IL-6 and TNF-α in gingival dialysate collected through a semi-permeable membrane from anaesthetised rats. Aside from the present findings, *Pg*-LPS and *Ec*-LPS can induce different effects on cytokines in specific groups of cells in the periodontal environment. For example, previous *in vitro* studies showed that *Pg*-LPS and *Ec*-LPS can increase the level of IL-6 in gingival fibroblasts^17,18^ and periodontal ligament cells.^22^

The *Pg*-LPS-induced increase in TNF-α production from gingival epithelial cells has been reported to correlate with the initiation of connective tissue destruction and bone resorption.^36,37^ TNF-α is thought to play both facilitative and suppressive roles in the pathogenesis of periodontal disease.^10^ The significance of the *Pg*-LPS-induced TNF-α observed in the present study in the pathogenesis of periodontal inflammation remains unclear. The present study showed that intragingival injection of *Pg*-LPS failed to alter gingival levels of IL-6, a pro-inflammatory cytokine, and did not induce gingival inflammatory infiltrate at the injection site. Repeated injection of *Ec*-LPS into rat gingiva is reported to induce inflammatory infiltrate at the injection site 5 days after the onset of treatment,^5^ but a single intragingival injection of *Ec*-LPS in the present study did not induce gingival inflammatory infiltrate at the injection sites. Taken together, these data suggest that (i) gingival inflammation with inflammatory infiltrate may not be induced immediately after introduction of *Ec*-LPS or *Pg*-LPS into the gingiva, and (ii) continued exposure to *Ec*-LPS may be necessary to induce inflammatory changes in the gingival tissue of experimental animals. Interestingly, a single injection of *S. typhimurium*-derived LPS into the rat gingiva induced significant periodontal inflammation with inflammatory infiltrate, apical migration of the junctional epithelium, interdental bone loss and activation of osteoclasts at the injection site 7–10 days after treatment.^6^ These studies clearly suggest that the effects of LPS on the induction of gingival inflammation with inflammatory infiltrate varies between bacterial species. In the present study, neither locally applied *Pg*-LPS nor *Ec*-LPS induced gingival inflammatory infiltrate at the injection site, but *Pg*-LPS and *Ec*-LPS clearly had distinct effects on the gingival extracellular levels of TNF-α.

In summary, the present study provides *in vivo* biochemical evidence that focal application of *Pg*-LPS, but not *Ec*-LPS, into the gingiva of rats transiently increases gingival levels of TNF-α without affecting IL-6. The present results suggest that TLR2 but not TLR4 expressed on gingival epithelial cells may mediate the *Pg*-LPS-induced transient increase in gingival TNF-α.

## Figures and Tables

**Table 1:** Mean basal values of cytokines in dialysate from the rat gingiva and the number of animals per group

Cytokines	Basal values/(pg·mL^−1^)	Number of animals
IL-6	389±76	24
TNF-α	Below detection limit	24

Basal values are expressed as the mean ± standard error cytokine level.
